# Transmission structured illumination microscopy with tunable frequency illumination using tilt mirror assembly

**DOI:** 10.1038/s41598-023-27814-x

**Published:** 2023-01-26

**Authors:** Krishnendu Samanta, Azeem Ahmad, Jean-Claude Tinguely, Balpreet Singh Ahluwalia, Joby Joseph

**Affiliations:** 1 Department of Physics, Indian Institute of Technology Delhi, New Delhi, 110016 India; 2 Department of Physics and Technology, UiT-The Arctic University of Norway, 9037 Tromsö, Norway; 3 Optics and Photonics Centre, Indian Institute of Technology Delhi, New Delhi, 110016 India

**Keywords:** Optics and photonics, Physics

## Abstract

We present experimental demonstration of tilt-mirror assisted transmission structured illumination microscopy (tSIM) that offers a large field of view super resolution imaging. An assembly of custom-designed tilt-mirrors are employed as the illumination module where the sample is excited with the interference of two beams reflected from the opposite pair of mirror facets. Tunable frequency structured patterns are generated by changing the mirror-tilt angle and the hexagonal-symmetric arrangement is considered for the isotropic resolution in three orientations. Utilizing high numerical aperture (NA) objective in standard SIM provides super-resolution compromising with the field-of-view (FOV). Employing low NA (20X/0.4) objective lens detection, we experimentally demonstrate $\sim$ (0.56 mm$\times$0.35 mm) size single FOV image with $\sim$1.7- and $\sim$2.4-fold resolution improvement (exploiting various illumination by tuning tilt-mirrors) over the diffraction limit. The results are verified both for the fluorescent beads as well as biological samples. The tSIM geometry decouples the illumination and the collection light paths consequently enabling free change of the imaging objective lens without influencing the spatial frequency of the illumination pattern that are defined by the tilt-mirrors. The large and scalable FOV supported by tSIM will find usage for applications where scanning large areas are necessary as in pathology and applications where images must be correlated both in space and time.

## Introduction

Breaking the diffraction limit^1,2^ of classical fluorescence microscopy in the last two decades revolutionized the biomedical studies and leading to a new field of research called ‘optical nanoscopy’^3,4^. In the rapidly progressing area of nanoscopy, structured illumination microscopy (SIM)^5,6,7^ appears as a significant widefield super-resolution technique where a series of structured patterns are employed to excite the fluorescent sample and corresponding raw Moiré frames are computationally processed to achieve around two-fold resolution enhancement over the widefield limit. In spite of offering relatively moderate resolution enhancement in comparison to the other optical super-resolution techniques, such as stimulated emission depletion (STED),^8,9^ stochastic optical reconstruction microscopy (STORM),^10,11^ photo activated localization microscopy (PALM),^12,13^ or ground state depletion (GSD),^14,15^ SIM has drawn considerable attention due to its high spatio-temporal resolution, low photo-toxicity, compatibility with common fluorescent labelling, efficient multi-colour imaging^16,17^ and so on. It is also considered a promising approach for the investigation of the sub-cellular dynamics of living cells^18,19^ for its requirement of less number of raw frames and low photon-dose. Although sinusoidal structured excitation pattern is the key feature to achieve the superior resolution imaging through SIM, it is no longer restricted to the periodic illumination patterns. Speckle-like^20,21^ random illuminations with blind reconstruction approaches are also successfully implemented for the SIM imaging. However, the random illumination methods offer super-resolution at the cost of low temporal resolution due to the requirement of a large number of frames ($\sim$100s). These methods wipe out the main theme of the periodic structured illumination imaging that minimizes the required number of frames 9(15) in 2D(3D) SIM cases,even less in certain cases^22,23^ reported recently. Hence, the efforts to further improve the resolution of SIM by maintaining the well-defined periodic illumination patterns are worthwhile.

In the conventional linear SIM technique, a single objective lens is employed for illuminating the sample as well as for collecting the fluorescence signal. Consequently, both the illumination and the detection optics are diffraction limited by the same objective lens, and the system is strictly restricted to offer $\le$ two-fold resolution improvement compared to the classical diffraction limit. In order to surpass the typical SIM resolution limit further, there is total internal reflection fluorescent (TIRF)-SIM^24,25^ where the high frequency interference pattern of evanescent waves illuminates the sample. However, the TIRF illumination is restricted to a thin optical section (<100 nm) and therefore deals with 2D imaging only. Besides, the saturation properties of the fluorescent materials are harnessed in the nonlinear-SIM^26–28^ approach which incorporates the contributions of multiple harmonics in super-resolution imaging. However, the requirement of high intensity to reach the operating saturation level of the fluorophores can provide photo-toxicity issues for the non-linear approach. Further SIM techniques based on different principles have also been reported, e.g., plasmonic^29,30^, proximity projection grating^31^ or photonic chip^32^. Each of these techniques requires sophisticated and dedicated photonic or plasmonic materials to manipulate the structured illumination pattern in the sample plane. In addition, specially designed tools are required for the phase-shifting and changing pattern orientation, e.g., thermo-optics for photonic chip SIM or galvo-scanning for the plasmonic SIM. In general, these techniques are complex due to their dependence on the dedicated material; which restricts the tunability of the illumination pattern frequency. In plasmonic SIM, the pre-calibrated array structures are fabricated according to the operating wavelength range. The specific structure selectively determines the orientation and the frequency of the illumination pattern, there is no tunability of the pattern. Similarly, in photonic chip SIM, the illumination pattern frequency is pre-decided by the angles of the available waveguide arms. Finally, conventional samples on microscope slides or coverslips do not work for these methods. It is required to prepare the sample on the surface of the designed material which is illuminated by the standing waves interference pattern in the evanescent regime, restricting these techniques to 2D-TIRF super-resolution.

In the race of achieving the best possible resolution, high numerical aperture objective lenses are typically utilized for the SIM imaging. The super-resolution image in SIM employing the high NA objective lens is obtained by compromising with the field of view (FOV). This limitation appears because of the inherent trade-off between the resolution and the FOV of the standard imaging system. Because of this dependence, the next level super-resolution techniques have been dealing with large FOV imaging while improving the resolution^33,34^. Recently, a theoretical approach^35^ was reported to overcome this limitation by decoupling the illumination path from the collection path using a transmission type tilt-mirror configuration. Nevertheless, the technique is not experimentally demonstrated for the bio-imaging application with scalable FOV and scalable resolution imaging. Herein, we demonstrate the experimental proof-of-concept of the tilt-mirror assisted transmission type SIM technique which possesses potential to exploit the high throughput SIM utilizing the free space optics in a cost-effective way. Liberating the illumination optics from the diffraction limit of the collection objective lens facilitates the full flexibility to play with the illumination configuration. This is, to the best of our knowledge, also the first experimental proof that describes it being possible to design the illumination for large FOV nanoscopy without guiding the light through any condenser lens^24,36^, waveguide chip^32,34^ or advanced materials^37,38^.

## Theory and optical setup

Decoupling the illumination from the detection optics in transillumination microscopy architecture paves the way to explore the versatile illumination modalities. In this work, we present a proof-of-principle experimental demonstration of transillumination SIM technique where an assembly of mini tilt-mirrors work as the illumination module. As the excitation path becomes independent of the collection path, the maximum frequency of the illumination pattern is no longer restricted by the detection objective lens.

**Figure 1:**
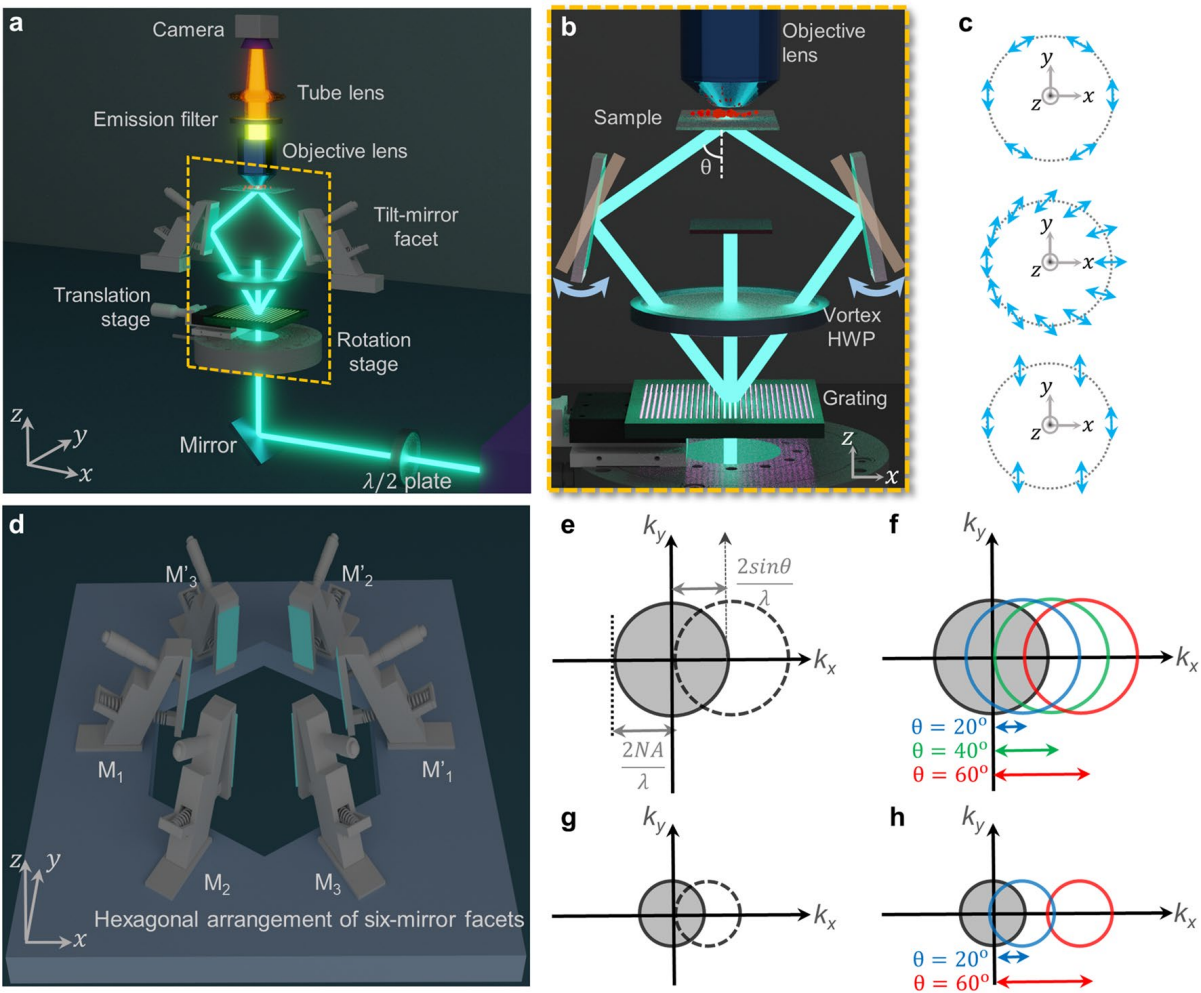
Concept of tSIM: (**a**) scheme of the experimental setup for one orientation; (**b**) tunable frequency illumination patterns are generated by a pair of mirror facets corresponding to low and high frequency illumination respectively; (**c**) scheme of polarization conversion of the interfering beams where top, middle and below panel corresponds to the state of polarization above, on and below the vortex half wave plate respectively; (**d**) hexagonal arrangement of mirror-facets for three orientation angles required for isotropic resolution enhancement; (**e**) resolution limit represented by the optical transfer function (solid circle), maximal shift (dotted circle) in conventional SIM offers two-fold resolution enhancement. (**f**) maximum spectral support of tSIM governed by with periodicity $\frac{\lambda }{{2\sin {\theta _1}}}$, tunable under different interfering angles $\theta$; (**g**, **h**) frequency support of a low-NA detection objective lens under similar illumination configurations, where tSIM support more than conventional 2X resolution enhancement of SIM. The 3D graphics shown in (a, b, d) are designed using a free and open-source software for 3D computer graphics, Blender (Version 3.2, https://www.blender.org/).

The graphical representation of the experimental setup is shown in Fig. 1a. The input laser is aligned parallel to the optical table and transmitted through a zero-order HWP to make the light polarized parallel to the optical table. It is then reflected by a $45^{\circ }$ inclined mirror towards vertical direction with polarization parallel to the mirror surface. To make the system compact, a physical grating is employed to split the input light, with the first order diffracted beams directed towards the optical axis (*z*) upon reflection by a pair of tilt-mirrors. The tilt angles of the oppositely faced mirror pair are adjusted in such a way that the reflected beams overlap in a small volumetric region to generate a sinusoidal interference pattern. At an instant, a pair of tilt-mirrors becomes functional for collecting the first diffraction orders from the grating. It is required to incorporate grating rotation and corresponding collection of the diffracted orders by other pairs of tilt-mirrors for isotropy. The purpose of the condenser lens is effectively fulfilled by the set of tilt-mirrors. This unique lensless illumination scheme possesses potential benefits in generating aberration free structured pattern which is homogeneous over the entire volume of interference. Besides, the area of illumination is no longer limited by the condenser lens; rather it is decided by the diameter of the interfering beams described in supplementary text T1. The sample is kept within the volumetric region to illuminate with the excitation pattern and a standard upright microscope is employed to collect the fluorescent signal from the other side of the sample. The maximum spatial frequency support of the transillumination SIM technique is determined by considering the resultant contributions from the detection and the illumination configuration,

1 $$\begin{aligned} {\mathbf {k}} = {{\mathbf {k}}_{det}} + {{\mathbf {k}}_{illu}} \end{aligned}$$

The detection system consisting of a lens with numerical aperture (NA) and emission wavelength $\lambda _{em}$ is restricted by the classical diffraction limit $\left| {\mathbf {k}}_{det}\right| = \frac{2\text{ NA }}{\lambda _{em}}$. On the other hand, the illumination frequency $\left| {\mathbf {k}}_{illu}\right| = \frac{2nsin\theta }{\lambda _{ex}}$ solely depends upon the interference conditions where $\lambda _{ex}$ is the peak excitation wavelength and $\theta$ is the semi interference angle between the interfering beams within a medium of refractive index *n*. Hence, the theoretical lateral resolution limit of the tilt-mirror based tSIM is determined as,

2 $$\begin{aligned} \Delta = \frac{\lambda _{em}}{2\left( \text{ NA }+\frac{\lambda _{em}}{\lambda _{ex}}n\sin {\theta }\right) } \end{aligned}$$

The semi-angle of interference ($\theta = 2\delta + \alpha$) is governed by the diffraction angle ($\alpha$) of the grating and the inclination ($\delta$) of the mirror with respect to the optics axis. Tuning the tilt angle of the mirror leads to the change in the interference angle and also in the pattern frequency. Hence, the technique is capable to generate the tunable frequency sinusoidal structured patterns depending upon the requirement of the illumination scheme. This particular characteristic is quite unique relative to the standard SIM technologies existing so far. The close-up view within the yellow-box in Fig. 1a is shown by Fig. 1b, representing the angle-tuning scheme for the illumination. The desired phase-shifting of the interference pattern in the sample plane is incorporated by shifting the physical grating along the lateral (along the grating period) direction with a single-axis piezo-translation stage. The piezo-stage is coupled with a motorized rotation stage which changes the orientation of the grating to incorporate isotropy in the illumination patterns. In order to create the illumination pattern of highest modulation depth, the polarization states of the interfering beams pairs are suitably converted before hitting the mirrors. Figure 1c represents the conversion scheme of the state of polarization: linearly polarized input (bottom panel), vortex half wave plate as polarization converter (middle panel) and azimuthally polarized output (top panel). The vortex HWP is suitably aligned to achieve the best possible interference pattern in each of the three orientations. Besides aligning the interfering beam pairs polarized parallel to each other, such conversion scheme efficiently utilizes the input power by minimizing the Fresnel reflection loss from the mirror surfaces. The description of the setup so far revolved around the excitation pattern along one orientation. The hexagonal arrangement of the tilt-mirrors are designed to create sinusoidal illumination along three different orientations for the isotropic resolution improvement. The hexa-assembly of the mirror facets are shown in the Fig. 1d, where (M$_{1}$, M$_{1}^{'}$), (M$_{2}$, M$_{2}^{'}$), (M$_{3}$, M$_{3}^{'}$) correspond to the three set of mirror-pairs. To change the illumination frequency, the equal tuning in the tilt-angles of each mirror-pair is done in such a way that the common volumetric interfering region shifts only along axial direction keeping the same lateral position. When the interference between the beam-pair occurs at a small angle ($\theta$), a large pattern period is created to provide low resolution gain whereas interference at high angle offers relatively better resolution. The resolution enhancement capability of the conventional SIM is presented by the gray circles in the Fig. 1e that represents around two-fold gain. On the other hand, the resolution improvement of the proposed tSIM is shown by the extension of blue, green and red coloured circles in the Fig. 1f corresponding to the semi-angle of interference $\theta = 20^{\circ }$, $40^{\circ }$ and $60^{\circ }$, respectively. The nitty-gritty details of the experimental setup is described in the supplementary text T2. Each frame is recorded with 300 ms exposure time and the recording of all images in three orientations takes 2700 ms in total. The rotation of the grating takes 750 ms for switching into two other orientations. Therefore, at present, the entire acquisition takes $\sim$3.5 s which is shown in the Table 1.

**Table 1:** Image acquisition speed of tSIM experiment.

Name of operation	Performed by employing the	Number of moves	Time per move (ms)	Gross time (ms)
Phase shifting	Piezo-translation stage	9	300	2700
Pattern rotation	Motorized rotation stage	2	375	750

The major advantage of the tSIM technique comes into play when the large field of view imaging is concerned, where a low-magnification/low-NA objective lens is utilized for the detection. Without modifying the detection passband (keeping the objective lens fixed), it is possible to incorporate illumination pattern of any arbitrary frequency with a maximum limit of $\frac{2}{\lambda }$ in the free space optics. Fig. 1g represents the resolution enhancement of a low-NA objective lens in traditional SIM case whereas Fig. 1h describes the tSIM case for the illumination patterns of variable spatial frequencies. Although an illumination pattern of very high frequency (compared to the low-NA passband limit) can be achieved to provide resolution far better than conventional SIM, intermediate frequencies from lower interference angles are required to prevent a gap. By tailoring the illumination patterns with the tilt-mirror assisted scheme, the technique is capable to fill the required frequency space and to offer resolution enhancement similar to a high-NA objective lens along with a large FOV supported by a low-NA detection objective lens.

**Figure 2:**
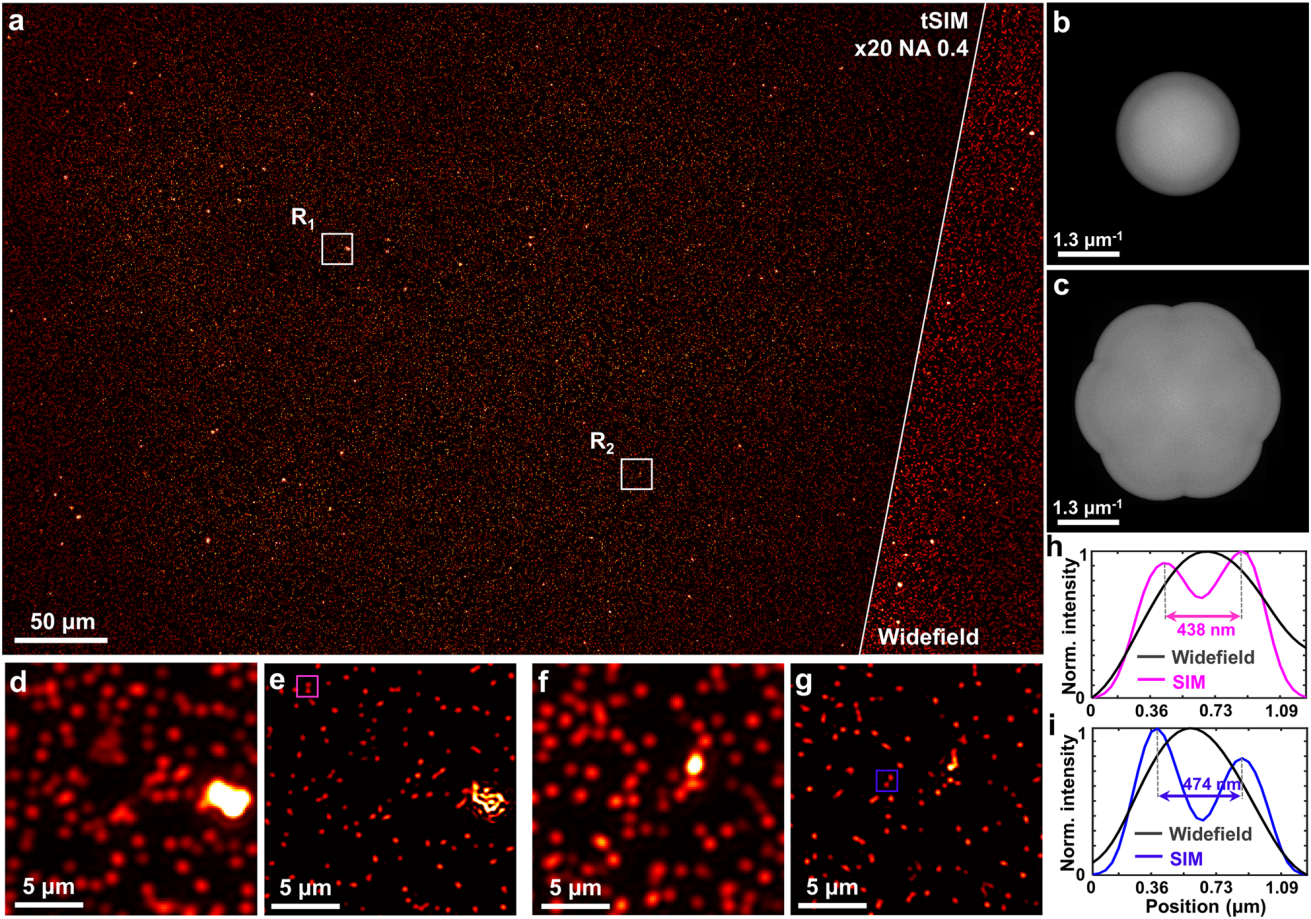
Experimental results of fluorescent beads: (**a**) single FOV tSIM reconstruction and widefield image of 200 nm fluorescent beads; (**b**) diffraction limited spectra; (**c**) reconstructed tSIM spectra; (**d**, **f**) magnified widefield images of white box in R$_1$ and R$_2$ regions, respectively; (**e**, **g**) closeup of the tSIM reconstruction in R$_1$ and R$_2$ regions, respectively; (**h**) intensity line-profiles of (**d**, **e**); (**i**) intensity line-profiles of (**f**, **g**).

## Results

### tSIM imaging with conventional illumination

In order to validate the super-resolution capability of the proposed tilt-mirror assisted structured illumination microscopy, we present experiments for polymer beads as well as a biological cell sample. For the proof-of-concept, first we demonstrate the experimental results for a scenario similar to the conventional SIM case where the illumination frequency lies within the passband limit of the detection system. A CW laser of wavelength 532 nm (DPSS 05-01, Cobolt Samba) is used as the excitation light source throughout the study.

**Figure 3:**
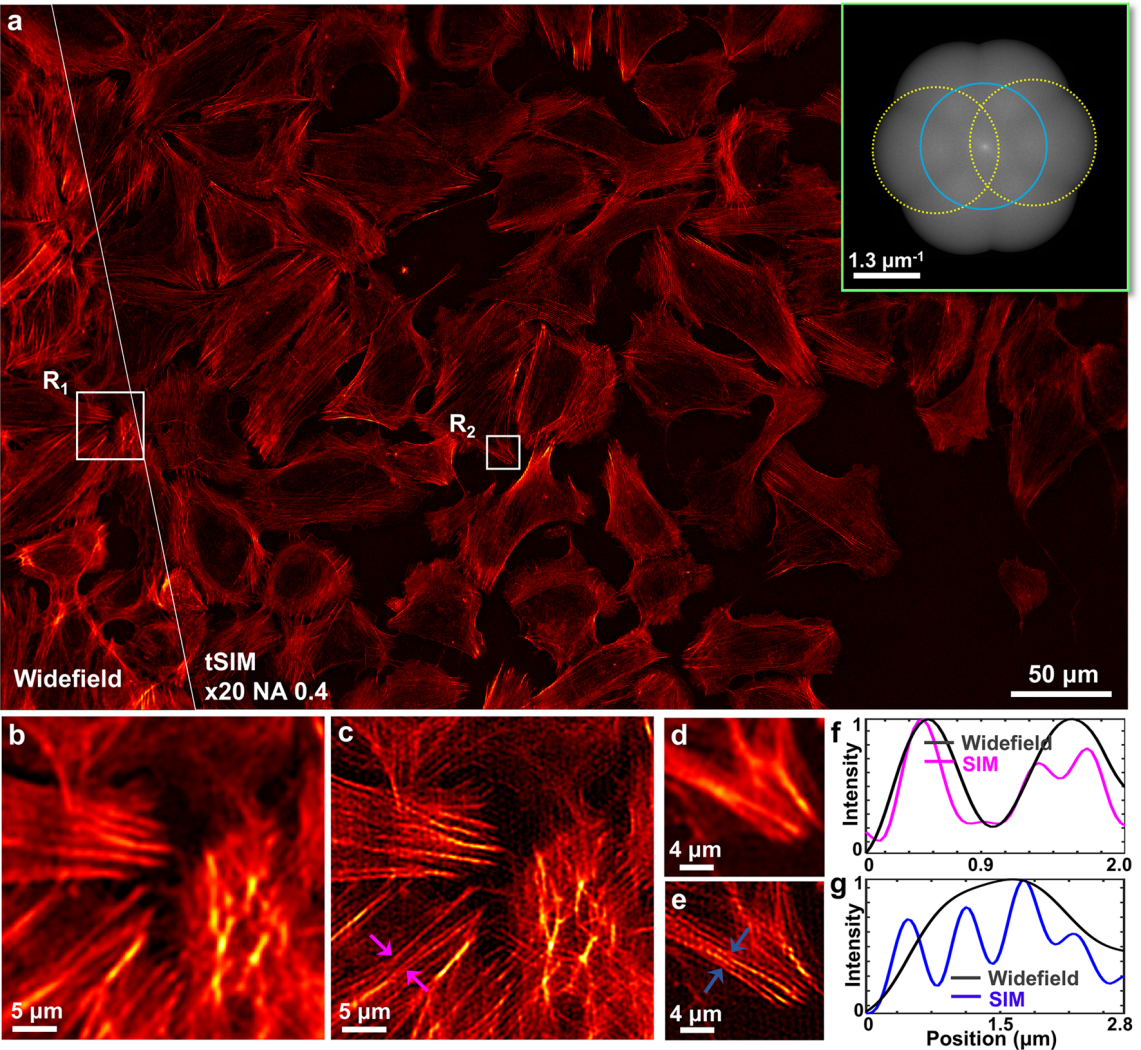
tSIM imaging of actin filaments in U2OS cells: (**a**) single FOV tSIM reconstruction versus diffraction limited image with the excitation/emission wavelengths of 532 nm/560 nm and inset shows spectra of the reconstructed image; (**b**, **d**) diffraction limited images of regions R$_1$ and R$_2$; (**c**, **e**) reconstructed tSIM image of regions R$_1$ and R$_2$ respectively; (**f**, **g**) intensity profile of a line marked between the arrowheads in (**c**, **e**).

The interfering beams are overlapped at the semi-angle of interference $\theta = 19^{\circ }$ to generate the sinusoidal illumination pattern of periodicity $\sim$778 nm. The interfering beams are of $\sim$1 mm in diameter to create the homogeneous illumination pattern over the entire area of illumination in the sample plane, which is sufficiently large relative to the FOV of the detection system. A standard upright fluorescence microscope is used as the detection system which consists of a low-NA objective lens (PLN 20X/0.4, Olympus), matched tube-lens (U-TLU, Olympus), fluorescence filter (550 nm long-pass, Semrock) and a digital camera with rectangular sensor (Hamamatsu ORCA spark, 1920$\times$1200 pixels). The sparsely distributed fluorescent nano-particles of 200 nm diameter (Bangslabs) are used as the fluorescent sample sandwiched between two cover-glasses. The excitation and emission peak wavelength of the fluorophore in the beads is 540 nm and 600 nm, respectively. Employing low-NA collection enables imaging with the large FOV of 0.56 mm width and 0.35 mm height (captured by rectangular sensor). Following the conventional SIM approach, three raw moiré frames are acquired by translating the physical grating with a step of 0.55 μm which leads to the phase-shifting of 0, 2$\pi$/3 and 4$\pi$/3 in the illumination pattern. The raw data for three different orientations are sequentially captured using three pair of tilt-mirrors in the hexagonal assembly. In total 9 frames (3 phases $\times$ 3 orientations) are computationally processed to achieve the final image. The open-source reconstruction algorithm ‘OpenSIM’^39^ is applied to process the raw tSIM data. The reconstructed image of the fluorescent beads and corresponding diffraction limited widefield image are shown in the Fig. 2a. The power spectrum of the widefield image is shown in the Fig. 2b, whereas the collected tSIM spectra are shown in Fig. 2c. The details of the separated spectral lobes are shown in the supplementary text T4. The magnified views of the white-box region R$_1$ are shown in Fig. 2d, e, which correspond to the widefield and reconstructed tSIM image, respectively. The intensity line profile across the beads in the region marked by the magenta-box is shown in Fig. 2h. The zoom-in details of another region R$_2$ is displayed by the Fig. 2f, g corresponding to the widefield and reconstructed image respectively. Figure 2i represents the line-scan profile across two fluorescent beads within the blue-box region. As the FOV is quite large, the analysis of several such zoom-in regions are shown in the supplementary text T3 to verify the homogeneous resolution enhancement over the entire FOV. Resolution enhancement is prominently visible from the intensity line profiles, where the continuous intensity distribution across two beads in the widefield image appears as two isolated particles after the tSIM reconstruction (Fig. 2(h,i)). The theoretical limit of the spatial resolution of the microscopy system is estimated to be 750 nm. It is thus confirmed that the separations above 430 nm are resolved by tSIM under conventional SIM conditions at a large FOV of 0.56 mm$\times$0.35 mm (restricted by the imaging sensor size).

**Figure 4:**
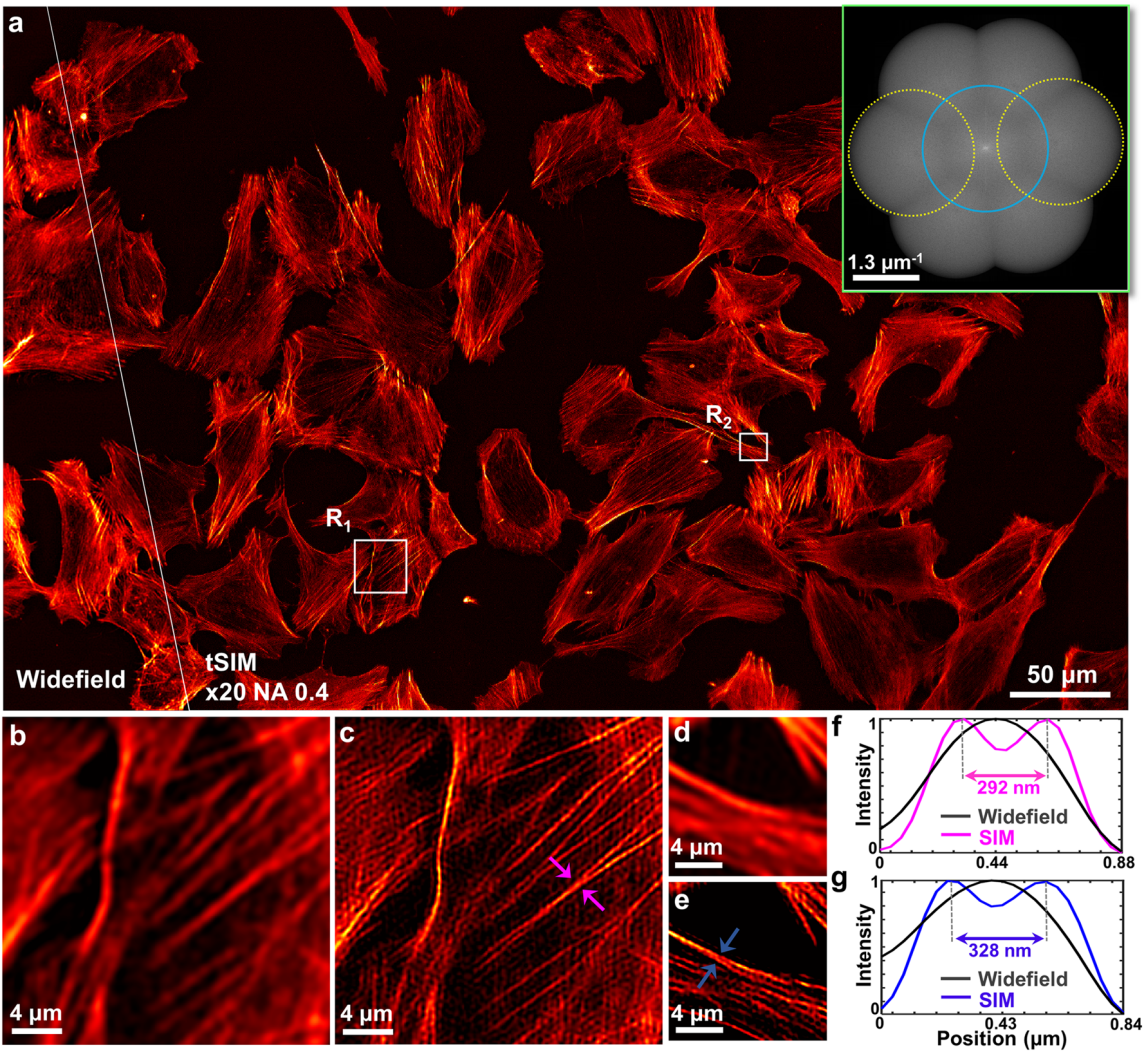
High-frequency tSIM imaging of actin-stained U2OS cell: (**a**) single FOV tSIM reconstruction and diffraction limited image with the excitation/emission wavelengths of 532 nm/560 nm and inset shows spectra of the reconstructed image; (**b**, **d**) widefield image of R$_1$ and R$_2$ regions respectively; (**c**, **e**) reconstructed image of R$_1$ and R$_2$ regions respectively; (**f**, **g**) intensity line profiles between the magenta and blue arrowheads.

**Figure 5:**
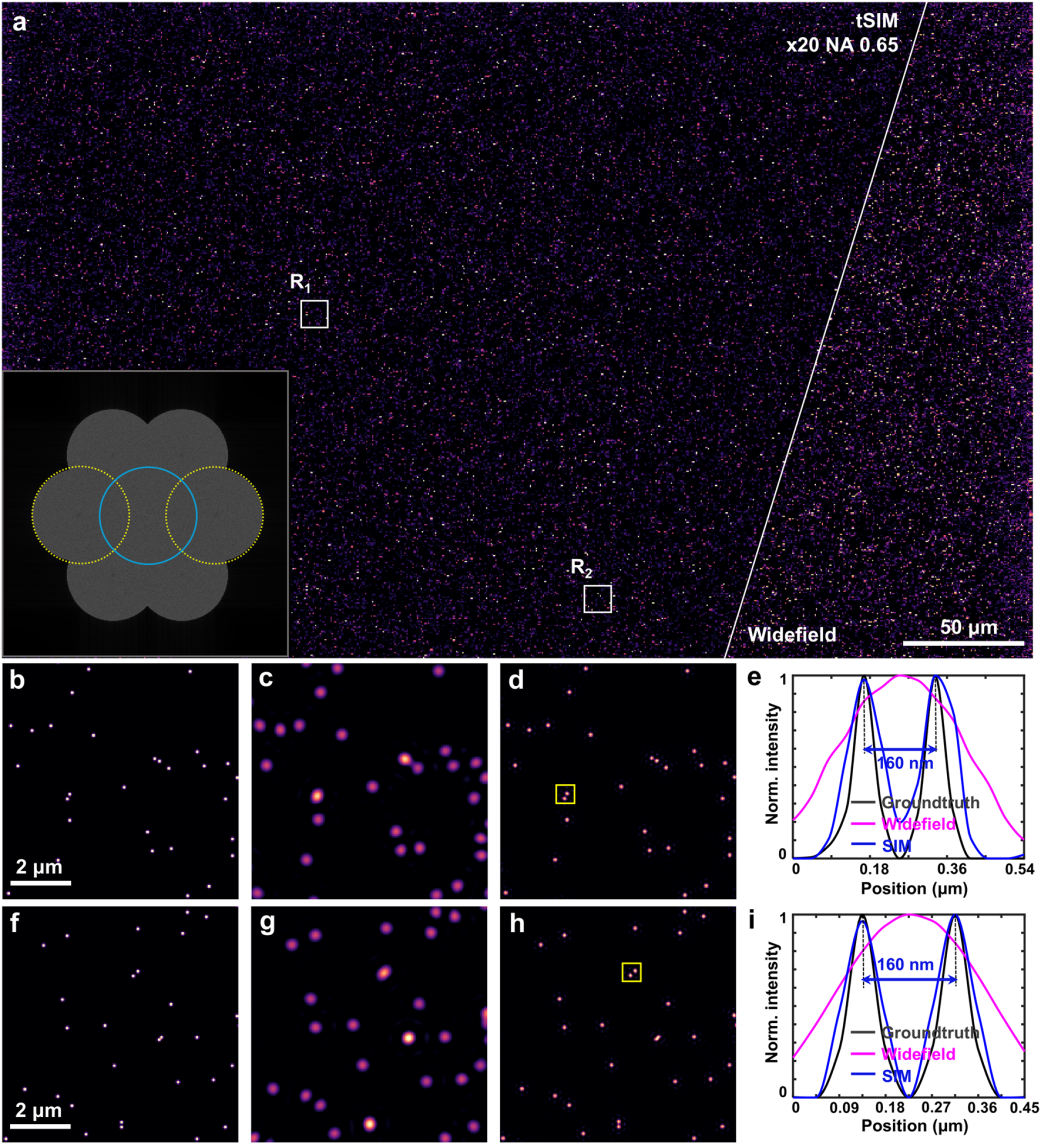
Simulation result of high-frequency tSIM imaging using synthetic fluorescent particles: (**a**) single FOV tSIM reconstructed image and widefield image using 20X/0.65 detection objective lens and inset shows spectra of the reconstructed image; (**b**, **f**) ground-truth object of R$_1$ and R$_2$ regions; (**c**, **g**) widefield image of R$_1$ and R$_2$ regions; (**d**, **h**) reconstructed image of R$_1$ and R$_2$ regions; (**e**, **i**) intensity line profiles within the yellow box.

Further, we explore the feasibility of the technique for a real application through a biological specimen. U2OS (human bone osteosarcoma) cells are seeded on glass coverslips, permeabilized, stained for actin filaments, and sealed with another glass coverslip on top. The 20X/0.4 objective lens is again employed here to simultaneously record more than 50 cells within the large FOV (0.56 mm width and 0.35 mm height). The sample imaging is performed in a region of interest where the concentration of the cells are moderate. The raw moiré frames are acquired under the identical imaging conditions as described for the case of the fluorescent beads. The reconstruction algorithm ‘OpenSIM’^39^ is implemented in this example as well, with the widefield and corresponding reconstructed tSIM image shown in Fig. 3a. The inset of Fig. 3a represents the spatial frequency spectra of the reconstructed tSIM image. The Fig. 3b, d represent the details of the widefield image from the white-box regions R$_1$ and R$_2$, respectively. The single FOV tSIM images corresponding to these regions are shown in Fig. 3c, e. The intensity line profile of Fig. 3c (marked by the magenta arrowheads) is plotted in Fig. 3f. Similarly, another line profile of Fig. 3e (marked by the blue arrowheads) is shown in Fig. 3g. The intensity line profile measurements of a few more regions are presented in the supplementary text T5. The features which appear as uniform distribution in the widefield image are resolved in the reconstructed image, where the spikes in the intensity profiles demonstrate the resolution enhancement of tSIM for the bio-samples.

### tSIM imaging with high frequency illumination

So far we have shown tSIM results with traditional illumination conditions. Now, we present an example where tSIM surpasses the conventional linear SIM resolution gain of two-fold, performed by implementing high frequency illumination with the pattern frequency outside the detection passband limit. The angle of the tilt-mirrors are tuned in such a way that the semi-angle of interference becomes $\theta$ = 35$^{\circ }$. This facilitates the generation of an interference pattern of 464 nm periodicity in the sample plane, which is below the diffraction limit of the detection system and thus fringes are not possible to observe. The raw moiré data is acquired by keeping the same detection objective lens (20X/0.4) as well as the previously mentioned imaging parameters. As before, 9 raw frames (3 phases $\times$ 3 orientations) were recorded in total. However, the conventional reconstruction approaches^39,40^ suffer to process the raw data in this particular case as the illumination frequency exists outside the passband support. This is overcome by utilizing a TIRF-SIM like reconstruction approach^39^. The reconstructed and corresponding widefield image is shown in Fig. 4a. The inset of Fig. 4a represents the frequency spectra of the reconstructed tSIM image. The off-center spectral components (marked with dotted yellow) are not overlapping which demonstrates the coverage to the broader spectral range due to high frequency illumination patterns. Figure 4b, d represents widefield image of two zoom-in regions R$_1$ and R$_2$ respectively. Their corresponding super-resolved images are shown by Fig. 4c, e. The intensity line profiles between the magenta arrowheads in Fig. 4b, c and the blue arrowheads in Fig. 4b, c are plotted in Fig. 4f and Fig. 4 g respectively. This leads to a resolution of $\sim$290 nm with the FOV of size 0.56 mm$\times$0.35 mm. Resolution enhancement of tSIM under different illumination configurations in terms of spectral support is presented in the supplementary text T6. A simulation study is presented by employing low magnification/ high NA objective lens and high tilt-angular illumination to demonstrate further resolution improvement with the same FOV. A 20X/0.65 objective lens is used for collection and the semi interference angle is kept $\theta$ = 70$^{\circ }$ which corresponds to an effective NA of 0.93. Random nanoparticle distribution of 80 nm diameter is chosen as the synthetic test target. The simulation result of single FOV tSIM reconstruction and diffraction limited image for 500 nm emission is shown in the Fig. 5a, inset shows frequency spectra of the reconstructed tSIM image. The Fig. 5b, f represent two zoom-in regions of the ground-truth object and Fig. 5c, g represent corresponding widefield images from the white-box regions R$_1$ and R$_2$, respectively. The single FOV tSIM reconstructed images corresponding to these regions are shown in Fig. 5d, h. The intensity line profile of Fig. 5d (marked by yellow square) is plotted in Fig. 5e. Similarly, another line profile of Fig. 5h (marked by the yellow square) is shown in Fig. 5i. This depicts the resolution down to 160 nm while maintaining the same FOV (0.56 mm$\times$0.35 mm) of 20X objective lens described in the previous cases.

### Scalable tSIM imaging

**Figure 6:**
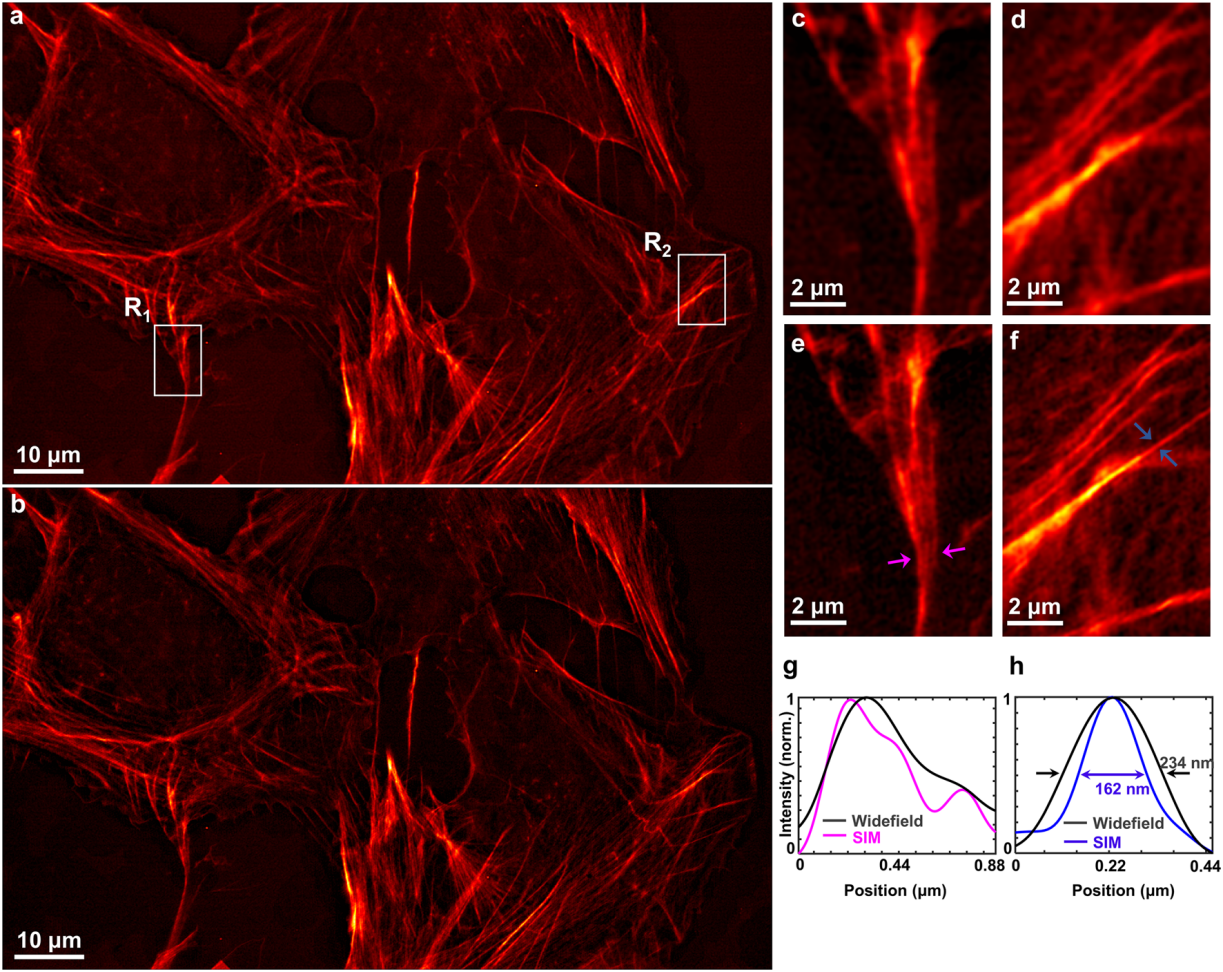
Experimental results of actin filaments of U2OS cell stained with alexa-fluor 532: (**a**) widefield image; (**b**) tSIM reconstruction image; (**c**, **d**) magnified widefield images of white box in R$_1$ and R$_2$ regions respectively; (**e**, **f**) closeup of the tSIM images in R$_1$ and R$_2$ regions respectively; (**g**) intensity line-profiles of (**c**, **e**) shown by magenta colour; (**h**) intensity line-profiles of (**d**, **f**) shown by blue colour.

All the previous discussion revolves around low-NA collection, large FOV imaging, tunable illumination patterns and corresponding super-resolution. However, it is mentioned that the overall resolution appears from the joint contributions of illumination and detection. Finally, the super-resolution capability of the tSIM utilizing the high-magnification/high-NA detection objective is demonstrated. We switch to 100X/1.3 oil immersion (Olympus) objective lens for the collection of the fluorescent signal while keeping the other imaging components (i.e. fluorescent filters, camera sensor) same as described for the previous cases. The same sample (U2OS cells stained with alexa-fluor 532) is used in this case also. The semi-angle of interference is kept $\theta$=$45^{\circ }$ that leads to generate interference fringes with spacing 376 nm respectively in the sample plane. This leads to a illumination case similar to conventional SIM with high-NA objective lens and the raw data are recorded in the same way described in previous cases. The widefield image is shown in the Fig. 6a whereas corresponding tSIM image is depicted in the Fig. 6b. Figure 6c, e represent the magnified views of the widefield image in the regions R$_1$ and R$_2$ respectively. The close-up view of the tSIM images are shown in Fig. 6d, f respectively. The intensity line profiles between the magenta arrowheads in Fig. 6c, e and the blue arrowheads in Fig. 6d, f are plotted in Fig. 6g and Fig. 6 h respectively.

Thus, employing high-NA detection objective lens improves the resolution of the detection system and applying tSIM further brings the resolution scale down to and $\sim$160 nm for $\theta$ = $45^{\circ }$. For a comparative analysis, we perform the experiment for the illumination angle $\theta$=$35^{\circ }$ and observe the resolution to be $\sim$190 nm and the corresponding results are described in the supplementary text T7. However, the super-resolution in employing high-NA (100X/1.3) detection objective lens appears at the cost of the FOV i.e. 0.11 mm$\times$0.07 mm which remains same for both the illumination cases with $\theta$ = $35^{\circ }$ as well as $\theta$ = $45^{\circ }$. The super-resolution capability of the tSIM with various illumination and detection configurations are presented in the Table 2 and some additional details are provided in the supplementary text T8.

**Table 2:** Super-resolution achieved by tSIM under different illumination-detection conditions.

Validation with the	Excitation/Emission (nm)	Interference angle ($\theta$)	Illumination effective NA	Detection objective	Field of view (mm$\times$mm)	Widefield resolution (nm)	tSIM resolution (nm)
Expt.	532/600	19$^{\circ }$	0.32	20X/ 0.4	0.56$\times$0.35	750	$\sim$430
Expt.	532/560	35$^{\circ }$	0.57	20X/ 0.4	0.56$\times$0.35	700	$\sim$290
Simulation	500/500	70$^{\circ }$	0.93	20X/ 0.65	0.56$\times$0.35	385	$\sim$160
Expt.	532/560	35$^{\circ }$	0.57	100X/ 1.3	0.11$\times$0.07	215	$\sim$190
Expt.	532/560	45$^{\circ }$	0.71	100X/ 1.3	0.11$\times$0.07	215	$\sim$160

## Conclusion

In conclusion, we demonstrate the proof-of-concept experimental results of tilt-mirror assisted transmission structured illumination microscopy (tSIM) which makes the illumination and the detection optics flexible to configure them independent to each other. The tilt-mirror facilitates to generate illumination patterns with tunable frequencies, which are of potential importance in order to provide the resolution gain depending upon the requirement. Employing the low-NA detection, a single large FOV image of size 0.56 mm$\times$0.35 mm (primarily limited by the sensor boundary) is obtained and we first demonstrate $\sim$1.7-fold resolution improvement (conventional SIM) through tSIM over the diffraction limit. The experimental verification is performed for both the fluorescent beads as well as for biological samples. Further by exploiting the high frequency illumination pattern (frequency peak outside the detection passband), a $\sim$2.4-fold resolution enhancement is achieved via tSIM with the same FOV. Moreover, we perform a simulation study to explore the possibility to further enhance the resolution by employing low magnification/ high NA collection objective and high tilt-angular illumination. It is observed that the traditional reconstruction fails to process the raw data for the cases when the illumination frequency lies outside the detection passband. We have implemented TIRF-SIM like reconstruction^39,41^ approaches for our reconstruction, however, there are possibilities to explore blind^42,43^ approaches which do not require the prior knowledge of the illumination patterns. Moreover, the deep-learning based^44,45^ algorithms might offer potential application in the tSIM reconstruction. The proof-of-principle experimental demonstration of tSIM for the fluorescent beads provides the perfect result. However, the reconstruction of the actin filaments contains some sort of honeycomb artifacts^46^ which is due to photo-bleaching and poor signal to noise ratio. Such reconstruction imaging artefacts are common in standard SIM image, however, the fluorescence is bright and stable for the beads sample and such artefacts were not observed. The feasibility of tSIM is justified with high-NA detection objective lens with the resolution down to $\sim$160 nm by compromising with the FOV.

The system is very compact in comparison to the typical SIM systems. The system at present takes $\sim$3.5 s for the entire acquisition. The speed is basically limited by the brightness of the sample, as acquisition time of 3 ms–10 ms is possible by using brighter fluorescent samples which possibly boost the system towards high-speed acquisition ($\sim$0.8 s) compatible for live-cell imaging over a large FOV (0.56 mm$\times$0.35 mm). The micrometer-screws described in the experimental setup for tuning the tilt mirrors were operated manually, the minor deviation of the pattern frequency due to slight deviation in micrometer-screw alignment was taken care during the (frequency estimation step) reconstruction process. However, by replacing these screw-heads with motorized versions would make the system fully computer controlled, accurate, faster and easy to operate. Employing high refractive index medium into the illumination configuration, sub-diffraction fringe patterns can be generated which is impossible to realize in free-space optics. This might have potential application to further enhance the resolution of tSIM. Different excitation sources can be coupled into the system to probe different fluorophores for the multi-colour SIM imaging^16,47^. Nevertheless, there is a scope to extend the proposed technique suitable for the 3D-SIM^17,48^ imaging by allowing the central beam into the tSIM architecture. In conventional SIM systems, the illumination and the collection is coupled and thus to obtain the small fringe period (high-spatial frequency), the two interfering beams should hit the edge of the back aperture of a given objective lens. Thus, every time a new objective lens is used for imaging, the optical arrangement must be changed to utilize the highest spatial frequency supported by the different objective lenses. Consequently, the commercial SIM system usually comes with a pre-defined single objective lens of high magnification and NA. This restricts the FOV of commercial SIM system to about $50\times 50$ μm^2^. The tSIM supports both the large and the user-defined FOV by simply swapping to another detection objective lens without influencing the illumination light path. The large FOV support of tSIM will find applications where high-throughput is essential such as in the histopathology^49,50^. Similarly, tSIM will benefit neuroimaging applications where data must be correlated both in space and time by imaging large areas^51^.

## Methods

### Preparation for fluorescent-beads sample

The coverslip was rinsed with diluted acetone and cleaned with deionized water. Next, it was treated with isopropyl alcohol solution and again cleaned with deionized water. Then, it was dried with the compressed nitrogen gas. A small (0.5 μl) amount of 200-nm diameter fluorescent bead solution (Suncoast yellow, FSSY002 Banglabs) was pipetted out and poured gently onto the cleaned coverslip. The coverslip was then kept at an inclined position and then allowed to dry completely. In this kind of drying, gravity pulls the droplet and the upper edge down during the liquid evaporation, which reduces the ‘coffee ring effect’. A tiny drop of glycerol was placed on another coverslip of bigger length; touching one edge of the coverslip containing beads with the bigger coverslip, the other edge was lowered slowly until it became completely flat on the glycerol. During this process, the sample side of the small coverslip faced towards glycerol so that the sample became sandwiched between two coverslips. The glycerol was allowed to spread out without pressing and the boundary between two coverslips was sealed with nail paint. After drying the paint, the sample was put under microscope for imaging.

### Cell culture and labeling

Osteosarcoma U2OS cells were cultured in Dulbecco’s modified eagle medium (DMEM, high glucose, Sigma-Aldrich) supplemented with 10% fetal bovine serum (Sigma-Aldrich) and 1% penicillin/streptomycin (Sigma-Aldrich) at 37 degree with 5% CO_2_. Cells (15–20% confluence) were seeded on #1.5 glass coverslips (22 mm$\times$22 mm, VWR) and incubated for 1 day before imaging. Then cells were washed in PBS, fixed with 4% formaldehyde (FA, ThermoFisher) in PBS for 10 min, washed three times in PBS, and followed with 0.1% TritonX-100 (Sigma-Aldrich) in PBS for 10 min for permeabilization, and washed three times in PBS. Cells were then incubated with Alexa Fluor^TM^ 532 phalloidin (1:50 in PBS, ThermoFisher) for 20 min at room temperature (RT) to label f-actin in the cells. Three times wash in PBS before mounting with ProLong$^{\mathrm {TM}}$ Glass Antifade Mountant (ThermoFisher) on glass slide/coverslips (24 mm$\times$50 mm, VWR) at RT overnight. Samples are then ready for the imaging.

### Experimental setup and data acquisition

The raw moiré frames were recorded using an upright modular microscope (BXFM, Olympus). The emission filter used for the imaging is the 550 nm long-pass fluorescent filter (Semrock). The rotation of grating was done using a motorized rotation stage (Holmarc). The physical grating was mounted on a single-axis translation stage and this stage including grating were attached together with the rotation stage. The linear piezo-translation stage (NF15AP25/M, Thorlabs) is operated along a particular direction to introduce suitable phase-shifting to the illumination pattern and a sequence of frames are recorded correspondingly. The acquisition process is repeated for three different orientations by revolving the grating around the optic axis using the rotation stage (MPR-SSHR-50, Holmarc).

### Image reconstruction

The computational image processing i.e. the reconstruction steps are performed only using MATLAB R2020b executed in a computer workstation running Windows 10 (Intel Xeon(R) W-2175 CPU, 2.50 GHz, RAM 64GB). First, the raw dataset is drift corrected through a custom-written algorithm. Then open-source package (Open-SIM) is employed to obtain the super-resolution image from the drift-corrected data. The associated code called TIRF-SIM in the same package is utilized for the reconstruction of data where the illumination frequency lies beyond the detection passband.

## Supplementary Information

Supplementary Information.

## Data Availability

The data that supports the findings of this study are available from the corresponding author upon reasonable request.
