# Supplementary material for: Transmission structured illumination microscopy with tunable frequency illumination using tilt mirror assembly
Source: Sci Rep. 2023 Jan 26;13:1453. doi: 10.1038/s41598-023-27814-x (PMC9879979; doi:10.1038/s41598-023-27814-x)
Supplement: Supplementary file 1 — Supplementary Information. [file 41598_2023_27814_MOESM1_ESM.pdf]

Transmission structured illumination microscopy with tunable frequency illumination using tilt mirror assembly: supplementary information

The supplementary document is associated with the parent article entitled 'Transmission structured illumination microscopy with tunable frequency illumination using tilt mirror assembly'. The supplementary information presents tilt-mirror angle tuning scheme, nitty-gritty details of the experimental setup, analysis of the tSIM result for fluorescent beads, power spectral support for the beads data, analysis of the tSIM result for the actins, spectral support of tSIM under different illumination configurations, scalable tSIM experiment with 100X/1.3 objective detection and $\boldsymbol{\theta}$=35° illumination, scalable super resolution achieved from the tSIM technique. It contains:

Supplementary texts (S1 - S8)
Supplementary figures (S1 - S7)
Supplementary table (S1)

SUPPLEMENTARY INFORMATION

Supplementary text 1: Tilt-mirror angle tuning scheme

TThe physical grating employed for diffraction contains 600 lines per mm i.e. the periodicity is 1.666-micron. The central order is blocked and the first diffraction orders (+1, -1 orders of equal intensity) are allowed to interfere and to generate the interference pattern at the sample plane. The first order diffracted beams come at an angle of (α) is 18.6° with respect to the optics axis (z) and the semi-angle of interference $\theta = 2\delta + \alpha$; where δ is the mirror tilt-angle with respect to the optic axis. The maximum possible δ is 30° with the current design, it can also be customized depending upon the requirements. So, the pattern frequency can be modified by changing the

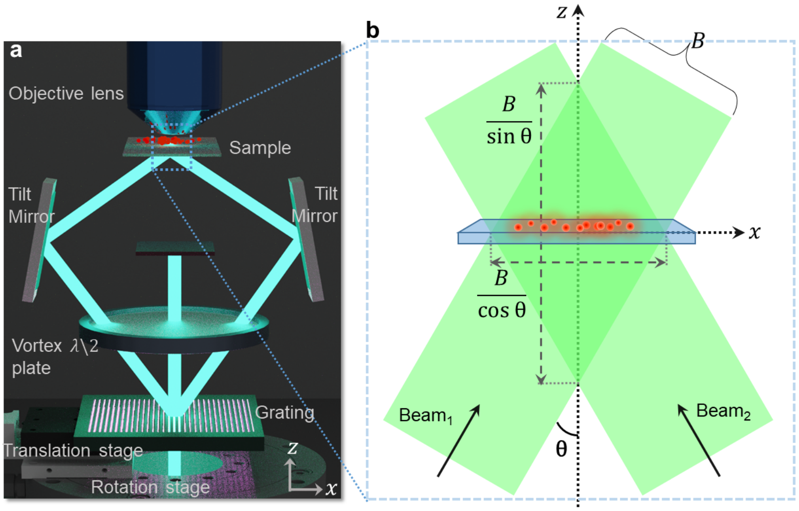

Fig. S1. (a) Side-view of the graphical experimental setup designed using a free and open-source software for 3D computer graphics, Blender (Version 3.2, https://www.blender.org/); (b) volumetric region of interference and span of illumination area.

diffraction angle (i.e. periodicity) of the physical grating or the tilt-angle of mirror. For a standard fixed grating, the tunability of the illumination appears only by tuning the mirror tilt-angle. The volumetric interference pattern is generated in region with $\frac{B}{\cos\theta}$ lateral and $\frac{B}{\sin\theta}$ axial spread. The span of the region depends on the beam diameter (B) and semi interference angle (θ).

Supplementary text 2: Nitty-gritty details of the experimental setup

The mirror assembly consists of six mirror facets arranged symmetrically around the central (z) axis. The mirror-mount is made of metallic aluminium (Al) and hence provide compactness and robustness to the illumination pattern. Each mirror in the assembly is inclined at an angle 60° w.r.t. horizontal direction. A micrometer screw-head is attached to each of the mirror facet, the

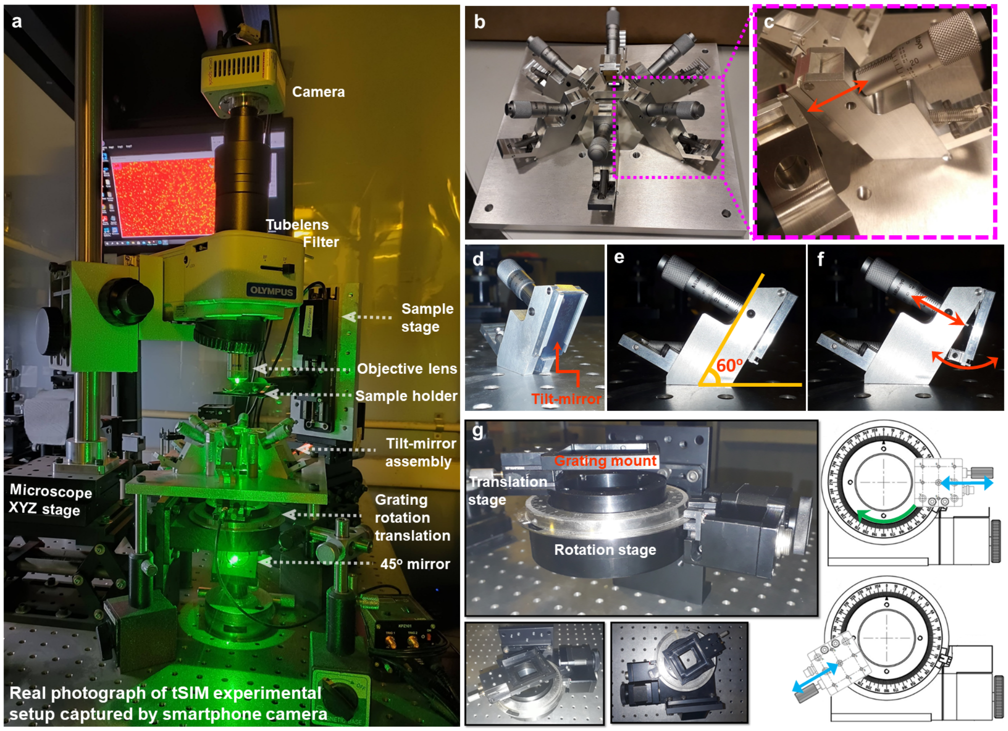

Fig. S2. (a) Photograph of the experimental setup; (b) metallic hexa mirror arrangement with the micrometer assisted angle tuning; (c) magnified view of a single tilt-mirror unit; (d) perspective view of single mirror unit; (e-f) angle tuning scheme of a single mirror facet; (g) coupled translation and rotation stage for the change of orientation and phase-shifting of the physical grating. All of these are the real photographs captured using smartphone camera.

linear movement of the screw head tunes and determines the tilt angle of that mirror. The whole system is built up vertically and imaging is done using a modular upright microscope kept on a xyz stage. The physical grating below the hexa-mirror assembly is mounted on single axis piezo translation stage which is further coupled with a motorized rotation stage. The combined opto-mechanical movements of the physical grating by the linear and rotation stages provide the desired phase-shifting and change in the orientation of the illumination patterns. The central beam blocker and vortex half wave plate are mounted in a 3D printed holder and placed between the physical grating and multi-mirror assembly. The sample mount is attached to a xyz stage to change the sample position according to the volumetric interference pattern.

Supplementary text 3: Analysis of the tSIM result for fluorescent beads

The tSIM imaging of the fluorescent beads under conventional case is presented earlier. The homogeneity in the large FOV imaging with better resolution is confirmed here with the magnified views of six different regions. The intensity line profiles across the beads in the selected box are

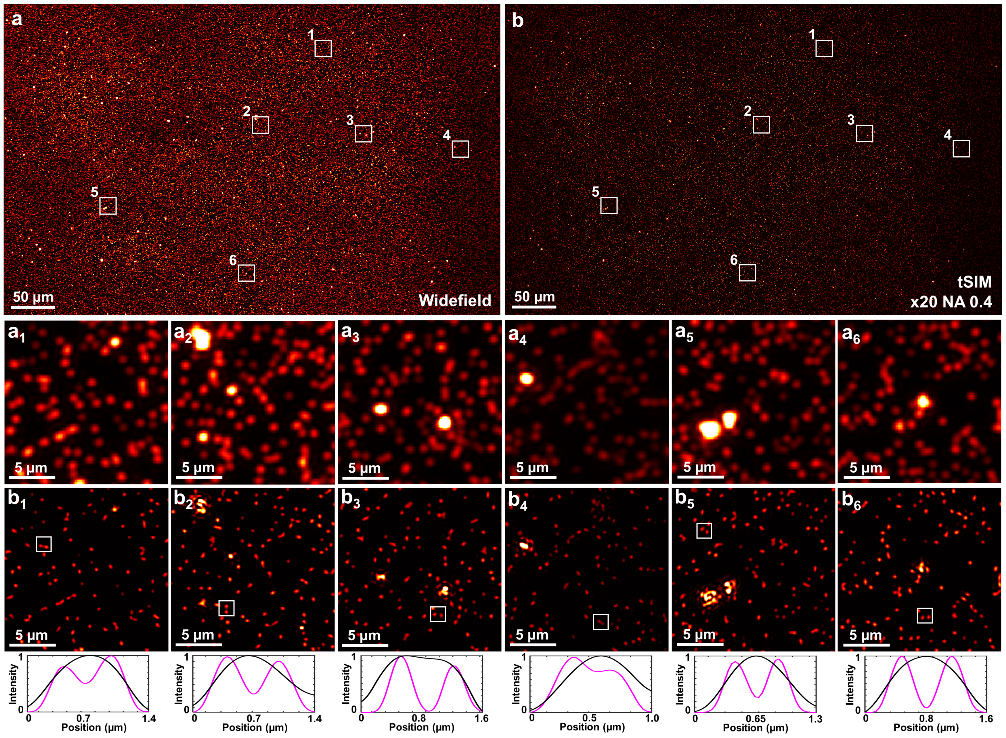

Fig. S3. Experimental results of fluorescent beads: (a) diffraction limited image; (b) SIM reconstructed image; (a_1-a_6) magnified views of (a) in different regions; (b_1-b_6) magnified views of (b) in different regions.

presented by the magenta and black coloured plots in the lower panel. This proves the resolution enhancement through tSIM in each of the zoom-in regions.

Supplementary text 4: Power spectral support for the beads data

The power spectra for the fluorescent beads data are shown here. The upper panel (figures a, b, c) show the off-center frequency components for three different orientations and the peak frequency is marked by the cyan-coloured circles. The lower panel (figures d, e) represent the

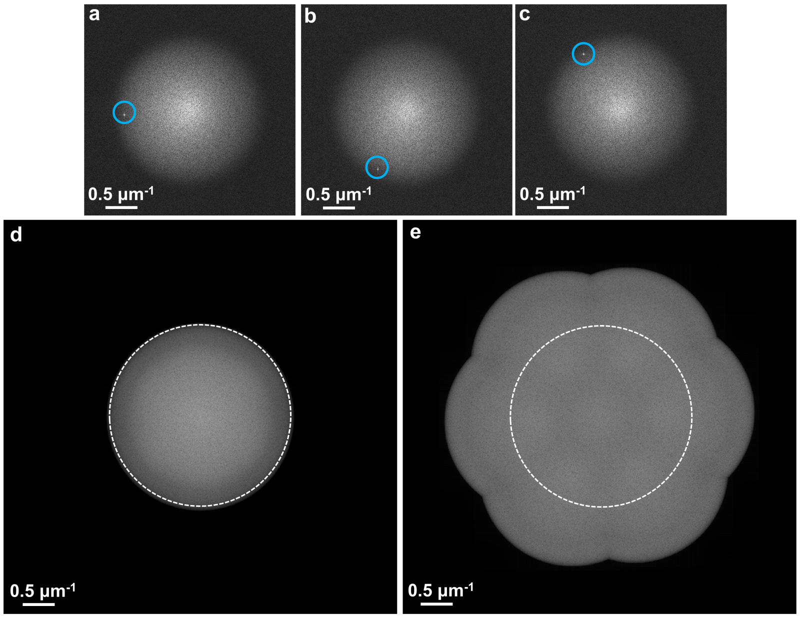

Fig. S4. 2D spatial frequency spectra of SIM data for fluorescent beads: (a-c) three separated spectral bands used for the reconstruction process where peaks corresponding to the illumination frequency and orientation are marked with the circles; (d) diffraction limited spectra; (e) reconstructed SIM spectra.

diffraction limited spectra as well as tSIM spectra respectively for the conventional illumination case ($\theta = 19°$). The white circles in both the cases correspond to the power spectral support of the diffraction-limited image.

Supplementary text 5: Analysis of the tSIM result for the actins

The tSIM imaging of actin filaments of U2OS cell under conventional SIM illumination scheme ($\theta = 19^{\circ}$) is previously presented and analyzed with some magnified regions. Here, the homogeneity in the large FOV imaging with better resolution is verified with the zoom-in views of a few more selected regions of the widefield as well as tSIM image, which visually confirms the actin filament in the reconstructed tSIM image to be nicely resolved.

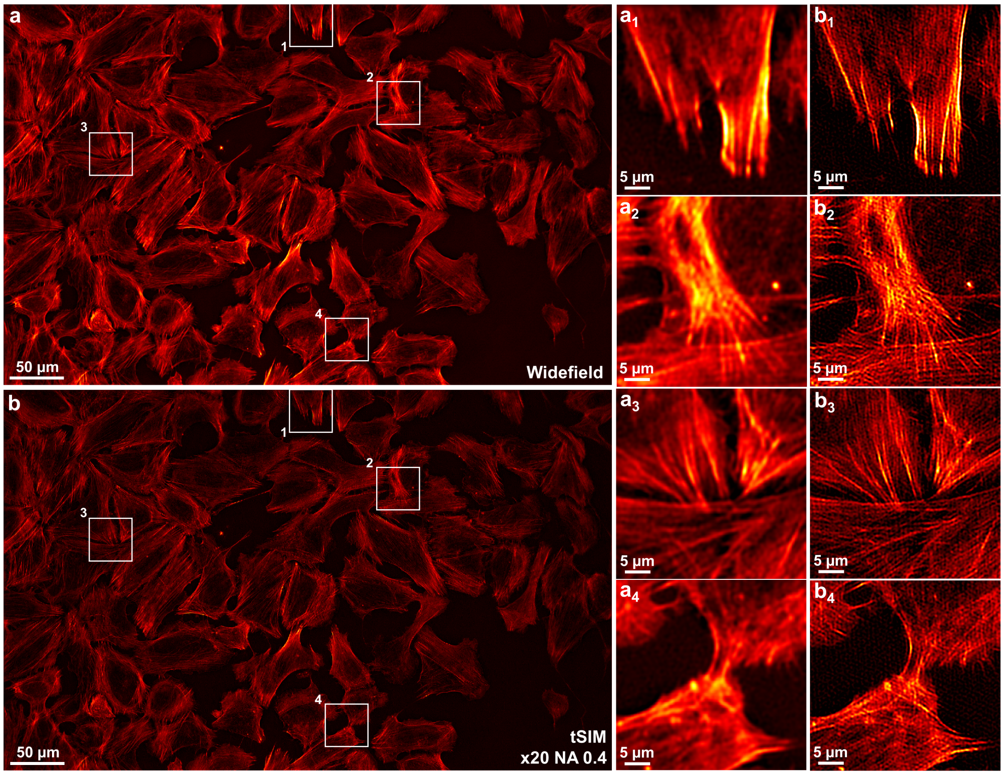

Fig. S5. Experimental results of actin filaments of U2OS cell stained with alexa-fluor 532 fluorescent tagging: (a) diffraction limited image; (b) SIM reconstructed image; (a_1-a_4) magnified views of (a) in different regions; (b_1-b_4) magnified views of (b) in different regions.

Supplementary text 6: Spectral support of tSIM under different illumination configurations

The spatial frequency support of the tSIM results for different illumination configurations using 20X/0.4 detection objective are shown here. The center circle (solid cyan colour) represents diffraction limited support of the detection system. The off-center circles (dotted yellow colour)

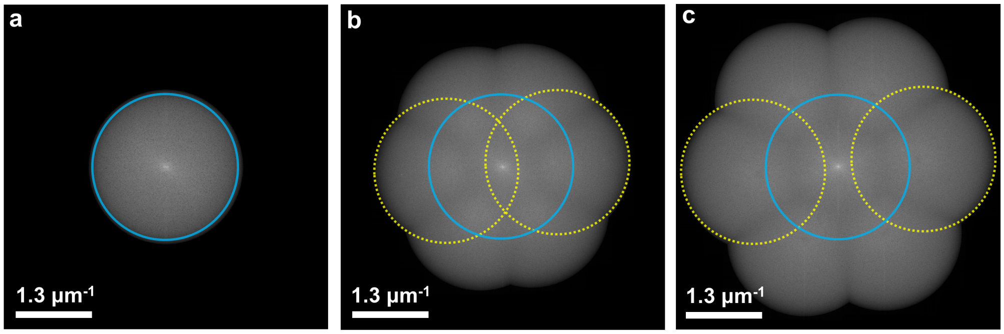

Fig. S6. Spatial frequency support: (a) diffraction limited image; (b) tSIM reconstructed image for conventional illumination; (c) tSIM reconstructed image for high frequency illumination.

corresponds to the high frequency spectral components. The tSIM spectra for two different illumination scenarios ($\theta = 19°$ & $\theta = 35°$) are shown for the comparative demonstration of passband extension due to frequency tuning. In first case ($\theta = 19°$), the illumination pattern frequency lies within the detection passband and the other case ($\theta = 35°$) deals with illumination pattern with frequency outside the detection passband.

Supplementary text 7: Scalable tSIM experiment with 100X/1.3 objective detection and θ=35° illumination

The scalable tSIM imaging experiment is performed with a lower semi-angle of interference. The angle of illumination in switched to θ=35° by tuning the tilt-mirrors and the 100X/1.3 oil immersion (Olympus) objective lens is used for the collection of the fluorescent signal in the detection side. The same sample (U2OS cells stained with alexa-fluor 532) is used also and all

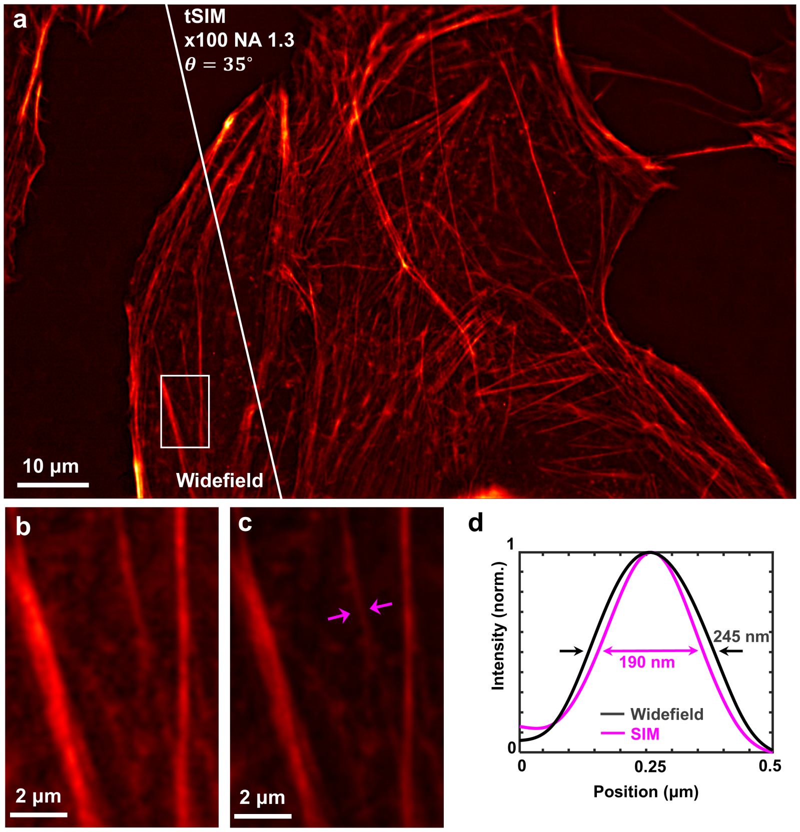

Fig. S7. Experimental results of actin filaments of U2OS cell stained with alexa-fluor 532: (a) single FOV tSIM reconstruction and widefield image using 100X/1.3 detection objective lens and angle of illumination θ=35°; (b) magnified widefield image of white box region; (c) magnified tSIM image of the same region; (d) intensity line-profile across a line between the magenta arrowheads in (b,c).

other image acquisition conditions and optical components (i.e. fluorescent filters, camera sensor) are kept same as the previous cases. The results are shown here which demonstrates the resolution goes upto ($\sim$190 nm) as lower angle (θ=35°) of illumination is employed here. However, the imaging FOV remains the same 0.11mm$\times$0.07mm as decided by the high NA detection objective lens (100X/1.3).

Supplementary text 8: Scalable super resolution achieved from the tSIM technique

The super-resolution capability of the tSIM with various illumination and detection configurations are already discussed in the main document. Some additional possibilities are shown here which

Table S1. Scheme of super-resolution using tSIM architecture

Collection objective lens	Illumination effective NA	Resolution gain in conventional SIM	Resolution gain in tSIM	FOV (mm×mm)
20X/ 0.4	0.4	350 nm (2-fold)	350 nm (2-fold)	∼0.5×0.5
20X/ 0.8	0.8	175 nm (2-fold)	175 nm (2-fold)	∼0.5×0.5
20X/ 0.8	0.9	†	165 nm (2.2-fold)	∼0.5×0.5
10X/ 0.4	0.9	†	215 nm (3.2-fold)	∼1.0×1.0
10X/ 0.25	0.9	†	243 nm (4.6-fold)	∼1.0×1.0

†Not possible using conventional SIM

can be achieved by using the specific objective lens and selective illumination. The numerical values presented in the above table are calculated numerically.
